# Comparative analysis of postoperative sexual dysfunction and quality of life in type a aortic dissection patients of different ages

**DOI:** 10.1186/s13019-021-01468-0

**Published:** 2021-05-01

**Authors:** Zeng-Rong Luo, Dong-Shan Liao, Liang-Wan Chen

**Affiliations:** Department of Cardiovascular Surgery and Cardiac Disease Center, Union Hospital, Fujian Medical University, Fuzhou, 350001 P. R. China

**Keywords:** Sexual dysfunction, Quality of life, Type a aortic dissection, Surgery, Different age

## Abstract

**Background:**

To compare postoperative sexual dysfunction (SD) and quality of life (QOL) in Type A Aortic Dissection (AAD) Patients of Different Ages.

**Methods:**

From January 2018 to December 2019, 204 AAD postoperative survivors in Union Hospital of Fujian Medical University were selected and were divided into young group (less than 50 years old) and elderly group (more than 50 years old). We evaluated SD according to the male International Erectile Dysfunction Index (IIEF-5) and female sexual function index (FSFI). The Short Form 12 Health Survey Questionnaire (SF-12) and Quality of Life Enjoyment and Satisfaction Questionnaire (Q-LES-Q) were used to investigate the QOL, Quick Inventory Depressive Symptomatology-Self Report (QIDS-SR) and the Beck Depression Inventory-II (BDI-II) to investigate depressive symptoms.

**Results:**

One hundred seventy-five patients completed all the questionnaire (85.8%). The total SD prevalence rate was 38.9% (68 cases), with 27.4% of the young (20 cases) and 47.1% of the elderly (48 cases). The age of non-SD and SD patients was 49.0 ± 11.5 and 56.9 ± 10.8 years, respectively (*P* = 0.03). Compared with non-SD patients, the total physical health of SD patients was significantly worse (*P* = 0.04), however, the mental health was not significantly worse (*P* = 0.77); the depressive symptoms did not expressed a significant difference between the SD and non-SD groups (QIDS-SR *P* = 0.15, BDI-II *P* = 0.06). Total physical health scores in the young SD group did not show significant better than elderly SD group (*P* = 0.24), however, total mental health scores showed significantly worse (*P* = 0.04), depressive symptoms scores were significantly higher (QIDS-SR *P* = 0.03, BDI-II P = 0.04).

**Conclusion:**

The postoperative AAD SD prevalence of elderly is higher than that of young, and the total physical health of SD patients is poorer than those without SD patients. The young SD patients did not show a significant higher physical health scores than the elderly SD patients, instead, the young SD patients were more psychologically affected than the elderly SD patients, whose mental health was worse, and depression symptoms were more obvious, suggesting that the factors affecting the QOL of postoperative SD patients are related to physical factors, but the young postoperative SD patients mainly affected by psychological factors.

**Supplementary Information:**

The online version contains supplementary material available at 10.1186/s13019-021-01468-0.

## Background

Acute aortic dissection (AAD) is an age-dependent, life-threatening cardiovascular disease associated with high mortality due to various fatal complications [1–7]. Studies have shown that long-term survival after initial treatment is good [8]. With many advances in surgical techniques in the past decade, efforts have been made to improve the survival rate after AAD surgery, and a lack of attention has been paid to improving quality of life (QOL). QOL covers physical, emotional, mental, professional and sexual aspects [9]. However, rare research has been conducted to evaluate the impact of changes in sexual function and emotion on the QOL of postoperative AAD survivors. Resuming sexual activity is one of the important factors for psychosocial recovery after cardiac surgery [10]. The aim of this study is to better understand the impact of sexual function status of postoperative AAD survivors at different ages on the QOL, and to provide possible suggestions for improving physical health and psychological health.

## Methods

### Research object

We totally distributed related questionnaires by mail or by outpatient appointments to 204 type A aortic dissection (AAD) survivors who successfully underwent “modified triple-branched stent graft implantation technique” surgery [11] in the Union Hospital of Fujian Medical University at the end of the 6th month after discharge from January 2018 to December 2019. The specific procedure was shown in the Supplementary Material: (1) During the regular process of cardiopulmonary bypass, block the ascending aorta, incise the ascending aorta, and perform proximal operations first, such as aortic valve repair, sinus reconstruction, ascending aortic artificial blood vessel replacement, etc.; (2) When the rectal temperature drops to 25 °C, antegrade selective cerebral perfusion is performed through the right axillary artery. Incision of the minor curvature of the aortic arch clearly exposes the openings of the three branches of the aortic arch and the true lumen of the descending aorta; (3) The circulation is stopped, the main part and branches of the modified triple-branched stent graft are respectively implanted into the true lumen of the descending aorta and corresponding three branch arteries on the aortic arch and released in turn; (4) Trim the stent and fix two branch stent graft; (5) Block innominate artery and insert the perfusion tube into the left carotid artery through the second branch stent graft, and perform bilateral antegrade cerebral perfusion; (6) Continuously suture the artificial blood vessel replacing the ascending aorta with the proximal end of the the modified triple-branched stent graft, and the whole body perfusion is restored before warming up and stopping cardiopulmonary bypass. This study was approved by the ethics committee of Fujian Medical University, China.

Inclusion criteria: (1) Patients who survived more than 6 months after being discharged from the hospital after successful AAD surgery; (2) 18 to 70 years old; (3) Fixed sex partners; (4) No mental illness, no cognitive dysfunction in reading, writing and communicating, able to read and comprehend the contents of the questionnaire; (5) Informed consent to this study, volunteered to participate in the study; (6) People who had regular sex before surgery.

Exclusion criteria: (1) Exclude those with mental illness, language communication barriers, dyslexia, and non-cooperation; (2) Exclude those with reproductive system diseases and organic diseases of other vital organs; (3) Exclude those with severe preoperative sexual dysfunction; (4) Exclude those who take drugs that seriously affect sexual function; (5) Exclude those who fail to fill in the questionnaire.

### Demographics

We collected the following demographic and clinical variables from all effective subjects: age, gender, chronic disease, marital status and the sexual partners situation.

### Research tools

#### Sexual dysfunction (SD)

We defined SD for men by using the International Index of Erectile Function-5 (IIEF-5): The International Index of Erectile Function (IIEF) was designed in 1997 by Rosen et al. [12], a doctor of American psychology. It originally contained 15 items to form IIEF-15. Rosen et al. [13] deleted 10 items after a series of analyses and the remaining 5 scoring items consist of IIEF-5 (including the confidence to maintain erection, the hardness of erection, the frequency of maintaining erection, the ability of maintaining erection, satisfaction with sexual intercourse). Because IIEF-5 and IIEF-15 have the same good evaluation effect and are highly recommended, it has become the most widely used and most authoritative questionnaire on male sexual function in the world. For the diagnosis of erectile dysfunction (ED) and the severity of ED, IIEF-5 can be used as an accurate and effective screening tool. A number of studies have shown that IIEF-5 also showed good reliability and validity in the Chinese population for the diagnosis of ED divided by 21 [13, 14]. Patients were scored according to IIEF-5 based on their erectile function in the past 6 months. IIEF-5 score ≥ 22 is classified as normal erectile function, ≤21 is classified as erectile dysfunction (ED), of which, scores 12–21 are classified as mild ED, 8–11 are classified as moderate ED, and ≤ 7 are classified as severe ED. The Female Sexual Function Index (FSFI) was used to define female SD which is a multi-dimensional scale used to assess female sexual function with score ranging from 2 to 36 [15]. SD for women was defined as obtaining a total FSFI score of < 26.5 [16].

#### Quality of life (QOL)

The Quality of Life Enjoyment and Satisfaction Questionnaire (Q-LES-Q) [17] were used to assess the QOL. The total score was calculated as a percentage of the total possible scores and reported as a score between 0 and 100. The lower the score, the worse the QOL. We also measured QOL using the Short Form 12 Health Survey Questionnaire (SF-12) [18], with scoring based on the RAN-12 score, which calculates the average score of physical and mental health among the general population in the United States, with an average score of 50 and a standard deviation of 10 [19]. Scoring higher or lower than 50 indicate better or worse function, respectively, as compared with the general population.

#### Depression

We used the Quick Inventory Depressive Symptomatology-Self Report (QIDS-SR) [20] to assess the severity of depressive symptoms, with a score ranging from 0 to 27. The severity of depression was also measured using the Beck Depression Inventory II (BDI-II) [21], with scores ranging from 0 to 63. For both assessments, higher scores represent more severe depressive symptoms.

### Statistical analysis

In this study, SPSS 20.0 statistical software was used to perform data statistical analysis. We performed normality test on measurement data firstly, and normally distributed data were expressed as mean ± standard deviation, analyzed with independent sample t test (corrected *P* value was used if the variance between groups was uneven); the enumeration data was described by percentage, the comparison of rates between groups was by Chi-square test. *P* < 0.05 indicated that the difference was statistically significant.

## Result

### General information of the research object

The response rate to the questionnaire was high, 85.8% of the participants completed the SD measurement, 92% completed the SF-12, and 97% completed the Q-LES-Q, QIDS-SR and BDI-II. Effective data analysis was performed on 175 patients who had completely answered all questionnaires, aged 23 to 70 years old, with an average age of 53.4 ± 8.5 years, 73 cases in the young group (mean age 43.4 ± 8.4 years, 41.7%) and 102 cases in the elderly group (mean age 59.4 ± 9.6 years, 58.3%); 144 cases were married or had a fixed sexual partner (82.29%), 31 cases were unmarried or divorced but had a fixed sexual partner; 119 cases of smoking (all men), 43 cases in the young group and 76 cases in the elderly group; of the total number of chronic diseases, 29 cases of diabetes, 143 cases of hypertension, 33 cases of dyslipidemia, 10 cases of coronary heart disease, 40 cases with renal dysfunction, 10 cases with COPD; 143 cases with medication (hypertensive, hypoglycemic and lipid-lowering drugs). The demographic characteristics are shown in Table 1. All patients underwent modified triple-branched stent graft implantation surgery about 6 months before starting the study.

**Table 1:** Preoperative characteristics

	Total	Young	Elderly	P1	SD	Non-SD	P2
Patients (n)	175	73 (41.7%)	102 (58.3%)	–	68 (38.9%)	107 (61.1%)	–
Age (y)	53.4 ± 8.5	43.4 ± 8.4	59.4 ± 9.6	0.000	56.9 ± 10.8	49.0 ± 11.5	0.033
Male (n)	148 (84.6%)	56	92	0.015	57	91	0.827
Chronic diseases							
Diabetes (n)	29 (16.6%)	5	24	0.003	19	10	0.001
Hypertension (n)	143 (81.7%)	49	94	0.000	62	81	0.010
Hyperlipidemia (n)	33 (18.9%)	6	27	0.002	17	16	0.098
Coronary heart disease (n)	10 (5.7%)	2	8	0.152	6	4	0.158
Renal dysfunction^a^ (n)	40 (22.9%)	12	28	0.087	21	19	0.044
COPD (n)	10 (5.7%)	2	8	0.152	5	5	0.457
Drugs (n)	143 (81.7%)	55	88	0.065	62	81	0.010
Smoking (n)	119 (68.0%)	43	76	0.029	59	60	0.000

^a^Defined as preoperative creatinine greater than 1.5 mg/dl

P1: *P* value of the youth group versus the elderly group; P2: *P* value of the SD group versus the non-SD group

*SD* Sexual dysfunction, *non-SD* Non-sexual dysfunction, *COPD* Chronic obstructive pulmonary disease

### SD prevalence

**Fig. 1:**
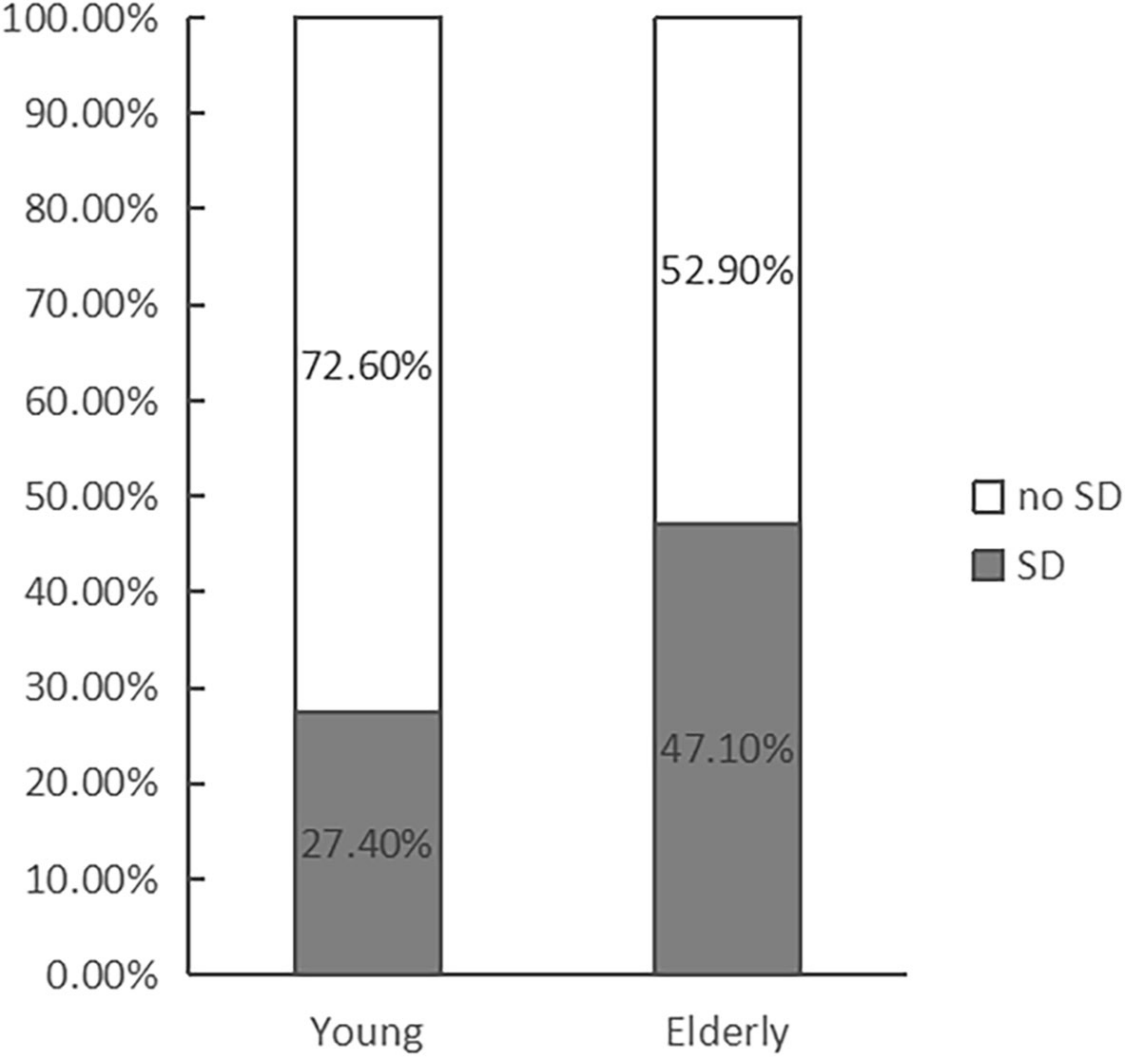
The percentage of postoperative AAD sexual dysfunction patients by different age groups

The total SD prevalence was 38.9% (68 cases), with the young prevalence of 27.4% (20 cases, 29% of the SD patients) and the elderly prevalence of 47.1% (48 cases, 71% of the SD patients) (Fig. 1).

### SD and QOL, depression

The age of non-SD and SD patients was 49.0 ± 11.5 and 56.9 ± 10.8 years, respectively. The total physical health of SD patients was significantly worse than non-SD patients (*P* = 0.04), including general health (*P* = 0.02), physical function (*P* = 0.04); However, the mental health of SD patients was not significantly worse than non-SD patients (*P* = 0.77), including vitality (*P* = 0.16), social function (*P* = 0.59), emotional role limitation (P = 0.59) and mental health (*P* = 0.75); the average depression score did not expressed a significant difference between the SD group and non-SD group (QIDS-SR *P* = 0.15, BDI-II *P* = 0.06) (Table 2). Total physical health (*P* = 0.24) in the young SD group did not show a significant higher scores than the elderly SD group, including physical function (*P* = 0.43), role limitation physical (*P* = 0.50), pain (*P* = 0.71) and general health (*P* = 0.64), however, the scores of total mental health in the young group was significantly lower than that of the elderly group (*P* = 0.04), including emotional role limitation (*P* = 0.04) and mental health (*P* = 0.03), the average depression score was significantly higher than that of the elderly group (QIDS-SR *P* = 0.03, BDI-II *P* = 0.04) (Table 3).

**Table 2:** SD is closely related to physical health

	Non-SD		SD		*P* value
	Mean	S.Dev.	Mean	S.Dev.	
Age (y)	49.0	11.5	56.9	10.8	0.03
QIDS-SR score	6.8	5.4	8.5	4.6	0.15
BDI-II score	6.5	6.8	9.7	6.6	0.06
Q-LES-Q score	78.2	10.6	70.4	12.3	0.08
SF-12					
Total physical health	60.0	10.8	49.6	11.8	0.04
Total mental health	68.0	12.2	60.3	10.9	0.77
Physical Functioning	60.0	11.0	50.2	11.1	0.04
Role limitation physical	62.3	11.5	59.5	9.6	0.36
Pain	65.5	9.5	54.3	13.3	0.49
General health	55.6	11.0	45.9	12.4	0.02
Vitality	53.5	14.8	48.3	9.9	0.16
Role limitation emotional	70.2	8.9	68.8	10.6	0.59
Social functioning	69.2	10.8	63.9	11.4	0.59
Mental health	62.3	10.8	60.1	12.1	0.75

*SD Sexual dysfunction, *non-SD* Non-sexual dysfunction, *S. Dev* Standard deviation, *Q-LES-Q* Quality of Life Enjoyment and Satisfaction Questionnaire-Short Form, *QIDS-SR* Quick Inven-tory Depressive Symptomatology-Self Report, *BDI-II* Beck Depression Inventory-II, *SF-12* Short Form 12 Health Survey

**Table 3:** SD in the youth group is closely related to mental health

	Young SD		Elderly SD		*P* value
	Mean	S.Dev.	Mean	S.Dev.	
Age (y)	45.4	11.9	60.7	11.9	0.02
IIED score	16.6	5.8	16.8	8.8	0.49
FSFI score	19.5	10.6	21.7	11.0	0.68
QIDS-SR score	11.2	4.8	8.3	5.6	0.03
BDI-II score	12.0	7.5	8.1	5.8	0.04
Q-LES-Q score	69.6	10.3	74.9	15.0	0.05
SF-12 score					
Total physical health	52.4	10.8	48.1	9.2	0.24
Total mental health	55.5	10.8	63.7	10.2	0.04
Physical Functioning	54.9	12.8	49.6	9.9	0.43
Role limitation physical	60.0	14.2	60.2	13.8	0.50
Pain	56.8	12.5	52.6	14.6	0.71
General health	49.6	10.4	44.4	10.0	0.64
Vitality	43.1	8.5	50.2	10.6	0.10
Role limitation emotional	61.8	10.5	69.8	12.8	0.04
Social functioning	60.8	11.8	64.2	9.9	0.36
Mental health	55.2	10.2	61.1	11.8	0.03

*SD Sexual dysfunction, *non-SD* Non-sexual dysfunction, *S. Dev* Standard deviation, *IIED* International Index of Erectile Dysfunction, *FSFI* Female Sexual Function Index, *Q-LES-Q* Quality of Life Enjoyment and Satisfaction Questionnaire, *QIDS-SR* Quick Inventory Depressive Symptomatology-Self Report, *BDI-II* Beck Depression Inventory-II, *SF-12* Short Form 12 Health Survey

## Discussion

Very few postoperative survivors will consult a doctor “when can I resume sex?” when discharged from the hospital, and few doctors will explain the precautions related to the patient’s postoperative sexual behavior. After all, this is an embarrassing question. Normal sexual function is a biological psychological process, usually has been found to be related to age, depressive symptoms, disease and hormone [22]. Some survey results show that SD after cardiac surgery is widespread [23]. SD can be multi-factorial in etiology with hormonal, anatomical, physiological, and psychological effects [9]. Previous studies have shown that sexual function changes after organ transplantation: For example, patients with end-stage renal failure undergoing kidney transplantation have been found to get improved sexual function [24–26]; however, despite the improvement in total quality of life (QOL) and physical function, SD seems to persist after heart transplantation. Tabler and colleagues found that the causes of SD after transplantation included drug side effects, fear of death during intercourse, depression, body-image concerns, uncertainty about the gender of the donor, and changes in roles and responsibilities in the family [27]. There is little information about the quality of sexual life and mood disorders of survivors after AAD surgery.

### SD prevalence

Our research shows that there is a certain prevalence of SD after AAD surgery (38.9%), and the prevalence of 47.1% in the elderly is significantly higher than 27.4% in the young. This may be because the majority of our research subjects are elderly people, most of whom are associated with chronic diseases. With age, the level of androgens in the body decreases. The decline of testosterone can reduce erectile response, sexual satisfaction and sexual frequency and also has a certain effect on ejaculation function [28]. In addition, the incidence of chronic diseases such as hypertension, diabetes, and dyslipidemia gradually increases with age, and the total number of combined chronic diseases is related to low interest in sexual activities or decreased satisfaction [29]. Therefore, instructing patients to actively treat various chronic diseases and strictly control blood pressure, blood sugar and blood lipid levels may be an effective way of improving the quality of sexual life in the cardiac rehabilitation program for patients after AAD surgery,.

### SD and QOL

#### SD is closely related to physical health

Our results show that the AAD postoperative SD patients showed obvious impairment of QOL, and we found that the impaired QOL was mainly manifested in physical health items, associated without depressive symptoms or clinically measurable mental health, which is consistent with the trend of QOL changes after heart transplantation [9]. The results of SF-12 show that the total physical health of AAD postoperative SD patients, including general health, physical function, and physical role limitations are lower than those of non-SD patients, but the total mental health items, including social function, emotional limitations and mental health, are not found significant difference between AAD postoperative SD patients and non-SD patients. Although this does not imply a causal relationship, this study suggests that AAD postoperative SD is more strongly related to physical health than mental health and may indicate that the occurrence of SD in this population is more likely to be a physical cause rather than a psychological cause. This is consistent with the results of previous studies: the study of kidney transplant recipients found a link between sexual function and physical health [30]. Wolpowitz and Barnard [31] found that after a heart transplant, a quarter of male subjects were unable to obtain or maintain an erection, despite their desire to have sex with their partners. Heart transplant patients found increased libido but impaired erectile function, resulting in a mismatch between libido and performance [32].

#### SD in the young group is closely related to mental health

In addition to aging and other confounding factors such as drugs and lifestyle factors, studies have shown that poor mental health is also related to SD. For example, there was a strong relationship between depression and SD [33]. Such patients mainly manifested as decreased libido, erectile function and decreased sexual activity [34, 35]. The relationship between mental health with AAD postoperative SD prevalence needs to be further clarified. Although self-reported depression and anxiety after AAD surgery were common [23], in our study, SD patients were not found a significantly closer relationship with mental health or depressive symptoms than non-SD patients. But interestingly, it was found that the impaired QOL of AAD postoperative SD patients in the young group is mainly reflected in the mental health items rather than physical health items. The mental health score of the young group was lower than that of the elderly group, and the depression symptom score was higher than that of the elderly group, although the physical health was not impaired as severe as that of the elderly group. These results indicate that although our previous study found that SD was generally more strongly related to psychological than mental factors after AAD surgery, the opposite is true for young SD patients, with more psychological factors than physical factors. Mild aerobic activity has been shown to promote physical and mental health and lower resting blood pressure after AAD surgery [36]. Clinicians will encourage postoperative AAD survivors to perform mild to moderate aerobic exercise when discharged from the hospital. Previous investigations have shown that postoperative AAD survivors have significantly reduced physical activity after surgery. Analyzing the reasons of increase in physical inactivity, it was unlikely due to impaired physical function after AAD, because most of our patients showed adequate physical function status [23]. This most likely results from psychological depression or anxiety or fear. Studies have shown that serious health conditions such as acute thoracic type A aortic dissection (ATAAD) are often associated with fear and can trigger a post-traumatic stress disorder [37], which is diagnosed more frequently in the case of female and younger patients [38, 39]. Our research also shows that the physical health of AAD postoperative SD patients in the young group did not show better than that of the elderly group, instead, the total mental health score is significantly lower than that of the elderly group, and the depression score is significantly higher than that of the elderly group. The questionnaire suggests that young patients think sexual activity is strenuous exercise and are fear of corresponding complications resulting from sexual activity. For example, due to the implantation of the stent, some young patients may worry about the displacement and shedding of the stent during sexual activity, and these psychological burdens will affect the patient’s sexual function such as erection and ejaculation to a certain extent and sexual dysfunction aggravate the impairment of mental health in turn. Therefore, when postoperative AAD survivors recovers the physical health, it is unnecessary to deliberately avoid sexual activities [40]. Health care providers should evaluate sexual health and encourage patients eliminate this unfounded psychological fear after AAD surgery. In recent years, many previous studies [41–44] have shown that providing sex education and counseling to patients and their spouses can improve the quality of sexual life of patients with cardiovascular disease and patients after cardiac surgery. It can be seen that it is necessary for postoperative AAD survivors to receive sexual health education provided by medical staff during hospitalization or discharge.

## Limitations

The sample size included is relatively small, especially in the young group. It is difficult to obtain accurate preoperative data about sexual function and quality of life (QOL). Selection bias may have occurred as patients were not randomized and the majority of patients were enrolled from outpatient clinic follow-up appointments or investigated by mailing questionnaires. Therefore, we might missed data of who died, who did not respond to emails or did not return to the outpatient clinic for review, but this only accounts for a small part of the survey subjects and does not seriously affect our survey results. Recall bias can also affect results as the data was acquired retrospectively. Some patients might have been experiencing SD, although we only include patients who deny preoperative SD. It was also a limitation that questionnaires were subjective assessment. Objective measures, such as activity, strength and ejaculation levels, in association with SD have not been well studied in the postoperative AAD population. Also, sex in the general population has appeared to be correlated with SD in heart transplant recipients [9]. This study could not rule out sex as a cause for SD, as it was not powered to address the effects of different sex groups on SD due to the small sample size.

## Conclusions

There is a considerable proportion of SD patiernts after AAD surgery, with higher ratio in elderly than young. SD seemed to linked to physical health rather than mental health of QOL. Interestingly, in contrast, young SD patients might experienced worse mental health rather than physical health of QOL.

## Supplementary Information

**Additional file 1.**

## Data Availability

Data sharing not applicable to this article as no data sets were generated or analyzed during the current study.

## Abbreviations

AAD: Acute aortic dissection
COPD: Chronic obstructive pulmonary disease
ED: Erectile dysfunction
IIEF-5: International Index of Erectile Function-5
FSFI: Female Sexual Function Index
SD: Sexual dysfunction
non-SD: Non-sexual dysfunction
S.Dev: Standard deviation
Q-LES-Q: Quality of Life Enjoyment and Satisfaction Questionnaire-Short Form
QIDS-SR: Quick Inven-tory Depressive Symptomatology-Self Report
BDI-II: Beck Depression Inventory-II
SF-12: Short Form 12 Health Survey
ATAAD: Acute thoracic type A aortic dissection
PTSD: Post-traumatic stress disorder
