# Supplementary figures and images for: Comparative analysis of postoperative sexual dysfunction and quality of life in type a aortic dissection patients of different ages
Source: J Cardiothorac Surg. 2021 May 1;16:117. doi: 10.1186/s13019-021-01468-0 (PMC8088030; doi:10.1186/s13019-021-01468-0)

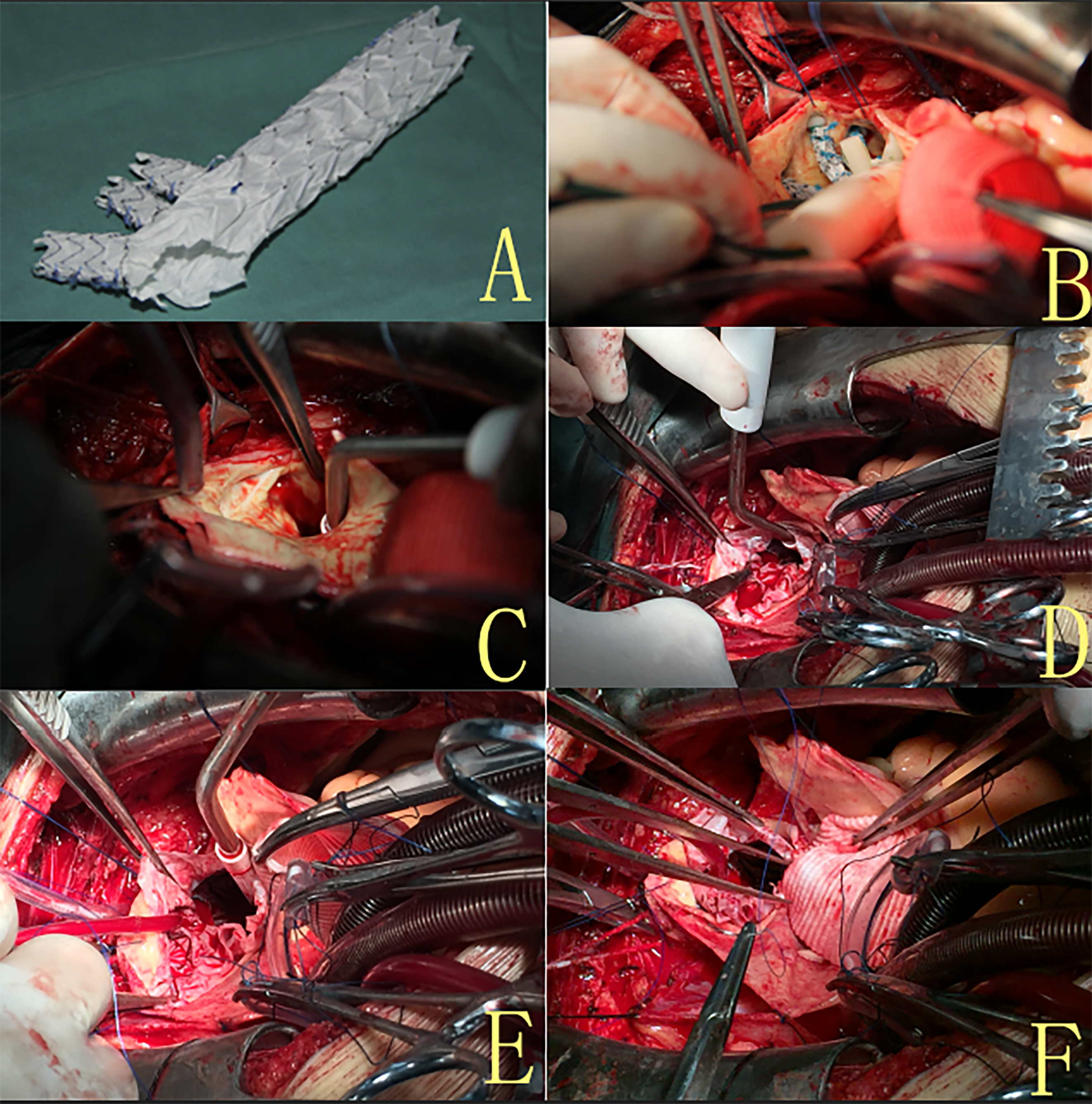

Supplement: Supplementary file 1 — Additional file 1. [file 13019_2021_1468_MOESM1_ESM.tif]
